# A study of the effect of half-day climatotherapy on changes in salivary cortisol levels

**DOI:** 10.1007/s00484-024-02698-2

**Published:** 2024-05-15

**Authors:** Hitomi Kanayama, Yukinori Kusaka, Hiroyuki Inoue, Takayoshi Hirai, Angela Schuh

**Affiliations:** 1 Division of Environmental Health, Department of International Social and Health Sciences, Faculty of Medical Sciences, University of Fukui, Eiheiji, Fukui Japan; 2 University of Fukui, Fukui, Japan; 3 Division of Generic and Global Studies, Faculty of Education, Humanities and Social Sciences, University of Fukui, Fukui, Japan; 4 Faculty of Nursing and Social Welfare Sciences, Fukui Prefectural University, Eiheiji, Japan; 5 Ludwig-Maximilians-University Munich, Munich, Germany

**Keywords:** Climatotherapy, Salivary cortisol, Circadian rhythm, Blood lactate, Physical activity, Mood

## Abstract

In our previous study setting, climatotherapy programme consisted of six sessions – four in the mid-mountain area and two in a flat park. For all sessions, the subjects underwent climatotherapy in the morning under slightly cool conditions. During each session, the subjects’ blood pressure, pulse rate, skin temperature, blood lactate, salivary cortisol and mood profile were recorded, and meteorological data were collected at the sites. We hypothesised that exercise habits, changes in mood profile and effective temperatures during the session, and physical exertion during the climatic terrain cure would affect salivary cortisol levels. Subjects were 30 (spring) and 29 (autumn). Multiple linear regression analyses were performed to examine the determinants of the change in salivary cortisol levels. In the mountain setting, salivary cortisol was elevated, even though the sessions took place in the descending phase of the circadian salivary cortisol variation; however, the post-session cortisol increase was not significant. Increased post-session salivary cortisol was significantly associated with female gender, older age, higher BMI, lower body fat, less daily physical activity, increased blood lactate, increased ‘Tension-Anxiety’ and ‘Depression-Dejection’ moods, and decreased ‘Anger-Hostility’ mood. The increase in cortisol may have been due to older age, a predominance of females, and the increased blood lactate due to the mountainous terrain. In the flat park, the significant decrease in postsession salivary cortisol was related to the descending circadian phase of circadian cortisol variation and the low physical demands of the sessions.

## Introduction

Salivary cortisol (sCort) is a steroid hormone released by the adrenal cortex in response to physical and psychological stress. sCort is known to have a circadian rhythm, with concentrations being highest immediately after waking and gradually decreasing until midnight (Hansen et al. 2006).

In a previous paper (Kanayama et al. 2024), we reported outcomes other than sCort for the climatotherapy programme conducted in 2015–2017. We hypothesised that individual biological characteristics, daily physical activity and net effective temperature (NET) at the climatotherapy sites, mood improvement and physical stress during climatotherapy would influence sCort levels in the subjects. In this short communication, we report the results of our analyses of sCort levels and associated factors.

## Materials and methods

In 2016–2017, saliva samples were collected with the Salivette® system before (approximately 9.30 am) and after (approximately 12.30 pm) climatotherapy sessions, frozen and immediately sent to a laboratory testing company (SRL, Inc., Japan) for analysis. The modified Missenard formula (Li and Chan 2000) was used to calculate the NET.

## Subjects

Thirty subjects participated in each of the 1st, 2nd, and 3rd sessions; due to grief caused by a family bereavement, one subject dropped out before the 4th session, leaving 29 subjects in each of the 4th, 5th, and 6th sessions.

All subjects were in a physical condition that did not affect their participation, but 8 were on antihypertensive drugs, 4 on antidiabetic drugs, 7 on cholesterol-lowering drugs, 1 on an anti-hyperlipidaemia drug, 4 on anti-ulcer drugs, 1 on a female hormonal drug, 2 on antigout drugs, 4 on antithrombotic drugs, 1 for sleeping pill, 1 for anti-anxiety drug. One subject taking a female hormonal drug which could influence salivary cortisol level was excluded from all statistical analyses.

The number of missing data points due to measurement error caused by insufficient saliva collection was 5 in session 1, 1 in session 2, 0 in sessions 3–5, and 1 session 6. A total of 108 sCort data points were available from the 1st, 3rd, 4th and 6th sessions in Yatsusugi Forest (YF) and 56 from the 2nd and 5th sessions in Fukui Prefectural General Green Center (GC).

### Statistical analysis

As the increase in subjects’ sCort levels was normally distributed for both at YF and at GC, paired t-tests were used to compare the increases in subjects’ mean sCort levels from pre- to post-session timepoints. Multiple linear regression analyses were then performed to examine the determinants of changes in subjects’ sCort levels. Biologically plausible predictors (gender, age, body mass index (BMI) and body fat percentage) were entered as independent variables using the forced entry method. Daily physical activity, number of exercise days per week, increase in blood lactate level from pre- to post-climatic terrain cure (ΔBLL), maximum pulse rate during the session as a percentage of estimated maximum heart rate (PR_max_/HR_max_ (%)), changes in T-scores on six scales of the Profile of Mood States (POMS) from pre- to post-session measurements, and NET during fresh-air rest cure (NET_rest_) were also entered as independent variables using a stepwise method. The inclusion criterion was *P* < 0.10 and the exclusion criterion was *P* > 0.20. Subjects with the missing values were excluded from the analyses. The significance level was set at 0.05. Multicollinearity was assumed if the variance inflation factor (VIF) was ≥ 10. Statistical analyses were performed using SPSS v28.0 (IBM Japan, Ltd., Japan).

## Results

The mean and standard deviation (mean ± SD) of sCort levels (µg/dL) were 0.137 ± 0.092 (pre-session) and 0.147 ± 0.140 (post-session) at YF, and 0.128 ± 0.089 (pre-session) and 0.103 ± 0.051 (post-session) at GC. At GC, post-session sCort levels were significantly lower (*p* = 0.008) than pre-session levels. At YF, although there was no statistically significant change (*p* = 0.391), sCort levels were higher after the session than before the session.

Table 1 shows the results of the multiple linear regression analyses. At YF, increased post-session sCort was significantly associated with female gender, older age, higher BMI, lower body fat, less daily physical activity, increased blood lactate, increased ‘Tension-Anxiety’ and ‘Depression-Dejection’ moods, and with decreased ‘Anger-Hostility’ mood. At GC, decreased post-session sCort was significantly associated with PR_max_ (%). There were no multicollinearities.

**Table 1:** Multiple linear regression analyses of changes in salivary cortisol levels from pre- to post-session at YF and GC

Site	*n*	Predictor variables	Adjusted R^2^	F	*P*	B	β	*t*	*p*	VIF
YF	108		0.363	7.782	< 0.001					
		(constant)				-0.719		-4.128	< 0.001	
		gender				0.148	0.626	3.157	0.002	6.601
		age				0.006	0.406	3.757	< 0.001	1.961
		BMI				0.015	0.348	2.957	0.004	2.333
		body fat percentage (%)				-0.009	-0.418	-2.402	0.018	5.095
		ΔBLL				0.019	0.361	4.524	< 0.001	1.070
		daily physical activity				0.044	0.318	2.904	0.005	2.020
		ΔPOMS_TA				0.005	0.278	2.897	0.005	1.543
		ΔPOMS_AH				-0.007	-0.360	-3.343	0.001	1.948
		ΔPOMS_D				0.007	0.290	2.641	0.010	2.023
GC	56		0.215	4.007	0.004					
		(constant)				0.067		0.579	0.565	
		gender				-0.017	-0.129	-0.490	0.626	4.894
		age				0.000	-0.045	-0.331	0.742	1.276
		BMI				0.000	0.006	0.029	0.977	2.702
		body fat percentage (%)				0.005	0.395	1.532	0.132	4.663
		PR_max_/HR_max_ (%)				-0.003	-0.443	-3.113	0.003	1.417

*B* unstandardised partial regression coefficient, *β* standardised partial regression coefficient, *VIF* variance inflation factor

*YF* Yatsusugi Forest, *GC* Fukui Prefectural General Green Center, *BMI* body mass index, *ΔBLL* increase in blood lactate level from before to after climatic terrain cure

*PR*_*max*_ maximum pulse rate during the session as a percentage of HR_max_, *HR*_*max*_ estimated maximum heart rate, *NET*_*rest*_ net effective temperature during fresh-air rest cure

*POMS* the Profile of Mood States

*ΔPOMS_TA* increase in T-score on the POMS ‘Tension-Anxiety’ scale from pre- to post-session

*ΔPOMS_AH* increase in T-score on the POMS ‘Anger-Hostility’ scale from pre- to post-session

*ΔPOMS_D* increase in T-score on the POMS ‘Depression-Dejection’ scale from pre- to post-session

*ΔPOMS_V* increase in T-score on the POMS ‘Vigor’ scale from pre- to post-session

*ΔPOMS_F* increase in T-score on the POMS ‘Fatigue’ scale from pre- to post-session

*ΔPOMS_C* increase in T-score on the POMS ‘Confusion’ scale from pre- to post-session

Variable Definition

gender (1: male, 2: female), daily physical activity (1: always, 2: sometimes, 3: rarely, 4: almost never)

As independent variables, gender, age, BMI and body fat percentage were entered using a forced entry method, and daily physical activity, number of exercise days per week, ΔBLL, PR_max_/HR_max_ (%), ΔPOMS_TA, ΔPOMS_D, ΔPOMS_AH, ΔPOMS_V, ΔPOMS_F, ΔPOMS_C and NET_rest_ were entered using a stepwise method

The inclusion criterion was *P* < 0.10 and the exclusion criterion was *P* > 0.20

A total of 108 salivary cortisol data measurements were available for YF (1st, 3rd, 4th, and 6th session), and 56 for GC (2nd and 5th session)

## Discussion

At YF, sCort was elevated, even though the sessions took place in the descending circadian phase (Hansen et al. 2006). This may be due to the older age of the subjects, the predominance of females, and the elevated blood lactate due to the mountainous terrain. The greater reduction in negative mood states may have suppressed the increase in sCort. There was no significant difference in sCort concentrations between pre- and post-session.

In contrast, there was no significant increase in blood lactate on the flat GC path (Kanayama et al. 2024). It is likely that the physically undemanding sessions in the tree-rich GC, in addition to the timing of the sessions during the descending phase of the circadian sCort cycle (Hansen et al. 2006), resulted in a significant reduction in sCort reduction.

The adjusted R^2^ was less than 0.50 at both sites, although there were no fixed assessment criteria. Further studies with larger numbers of subjects are needed.
